# Functional Fitness and Quality of Life among Women over 60 Years of Age Depending on Their Level of Objectively Measured Physical Activity

**DOI:** 10.3390/ijerph16060972

**Published:** 2019-03-18

**Authors:** Agnieszka Nawrocka, Jacek Polechoński, Wiesław Garbaciak, Władysław Mynarski

**Affiliations:** 1 Department of Physical Activity and Health Prevention, The Jerzy Kukuczka Academy of Physical Education in Katowice, 43-512 Katowice, Poland; j.polechonski@awf.katowice.pl (J.P.); w.mynarski@awf.katowice.pl (W.M.); 2 Department of Theory and Methodology of Physical Education, The Jerzy Kukuczka Academy of Physical Education in Katowice, 43-512 Katowice, Poland; w.garbaciak@awf.katowice.pl

**Keywords:** WHOQOL, senior fitness test, exercises, Actigraph, accelerometry, elderly, older adults, physical fitness

## Abstract

The aim of this study was to identify the differences in functional fitness and quality of life among women over 60 years of age depending on their level of objectively measured physical activity (PA) according to Global Recommendations on Physical Activity for health. The study used a cross-sectional design with 213 female volunteers over 60 years of age. Physical activity was monitored for 7 days of the week using an Actigraph Gt3X monitor. The Senior Fitness Test battery and hand-grip strength tests were performed to assess functional fitness. Quality of life was self-reported using a short version of the World Health Organization Quality of Life questionnaire WHOQOL-bref. Women who met the PA recommendations achieved slightly better results in most functional tests and in all domains of quality of life. Significant differences were found in the upper body strength, dynamic balance, and social relationships domains of quality of life. Physical activity programs developed on the basis of World Health Organization (WHO) recommendations have the potential to improve functional fitness and quality of life. However, further experimental studies in this area are required.

## 1. Introduction

Physical activity is one of the most important health behaviors, preventing many age-related problems. It is associated with successful aging [1,2,3]. There is a lot of research evidence that physical activity reduces the risk of many noncommunicable diseases, including cancer, coronary diseases, diabetes, and pulmonary diseases [4,5,6]. Regular physical activity can also improve mental health, cognitive functions, and overall well-being [7,8,9,10]. Many studies have confirmed that people that are more physically active have a lower rate of overall mortality [11,12].

Therefore, a minimal level of physical activity is promoted by many international and national organizations for maintaining the health and functional capacity of societies. The best known and documented are the Global Recommendations on Physical Activity for Health developed by the World Health Organization (WHO) [13]. In these guidelines, an accumulation of at least 150 min of moderate physical activity (MPA) or at least 75 minutes of vigorous physical activity (VPA) during the week is considered as a bare minimum for adults and older adults. It is recommended to increase physical activity up to 300 min of MPA or 150 min of VPA per week to achieve more health benefits. 

Considering the fact that the world’s population is aging, special attention should be paid to the health behaviors of older people. The participation of seniors in physical activity has a beneficial effect on functional ability and reduces the risk of functional limitations [14,15]. It allows them to maintain independence and keep self-reliance, which is very important for good quality of life. Therefore, the monitoring of percentages of adults and older adults who meet recommendations with respect to physical activity for health is an important and current research problem [16,17,18].

The relationship between various parameters of physical activity and functional fitness among older adults was confirmed in the previous studies [19,20]. Some researchers also emphasized the association between physical activity and quality of life among middle-aged and older adults [21,22,23]. However, the physical activity was measured using subjective self-reported assessment methods (questionnaires). It was confirmed that self-reported physical activity is often overestimated [24,25].

Therefore, the aim of this study was to identify the differences in functional fitness and quality of life among women over 60 years of age depending on their level of objectively measured physical activity according to the Global Recommendations on Physical Activity for health.

## 2. Materials and Methods 

### 2.1. Participants

This study was approved by the ethical committee of the Jerzy Kukuczka Academy of Physical Education in Katowice. The study used a cross-sectional design with 213 women over 60 years of age. 

All participants were volunteers from clubs and universities for seniors from urban regions near Katowice, Poland. The research project was promoted in all clubs and universities for seniors located in Katowice and adjacent cities through promotional posters and leaflets. People interested in participation in the research were invited to meetings, where the aim of the study, protocol, and research tools were presented. The inclusion criteria for participation in the study were as follows: age over 60 years, female gender, and agreement to participate in the full study protocol (functional fitness assessment, weekly monitoring of physical activity using the Actigraph monitor, and quality of life assessment using questionnaire). Participants with health conditions not allowing fitness test participation, and/or with any acute illnesses and musculoskeletal injuries were excluded. The total number of participants was 213, after including only women who met the inclusion criteria, had undergone the full research program, and had complete data from all measurements according to adopted criteria described in the methodology section. Women with missing data were excluded from analysis. More detailed participant characteristics are presented in Table 1.

### 2.2. Physical Activity Assessment

Physical activity was recorded for seven consecutive days using a tri-axial accelerometer Actigraph Gt3X (ActiGraph, Pensacola, Florida) worn on the waistband. Data from Actigraph were processed using the following settings:

- Acceleration were accumulated from three axes (vertical, medio-lateral, anterio-posterior) and combined into a vector magnitude score (VM);
- A valid day was defined as a minimum of 10 h of wear time;
- Data were integrated into 60-second epochs and expressed as counts per minute (cpm);
- Non-wear time was defined as 60 consecutive minutes of 0 cpm, with allowance for 1–2 minutes of counts 0–200 cpm (Troiano algorithm modified for VM data) [26,27];
- The raw data from Actigraph were analyzed using Actilife v5 software (ActiGraph, Pensacola, FL, USA) and three levels of physical activity were identified according to following threshold counts values: 200–2689 cpm for light physical activity (LPA), 2690–6166 cpm for moderate intensity of physical activity (MPA), and more than 6167 cpm for vigorous intensity of physical activity (VPA) [28]. Physical activity was assessed according to Global Recommendations on Physical Activity for Health developed by World Health Organization (WHO) [13]. Therefore, women who accumulated at least 150 min of moderate physical activity or at least 75 min of vigorous physical activity or combination of moderate and vigorous activity during the week were considered to be sufficiently physically active.

### 2.3. Functional Fitness Assessment

The Senior Fitness Test was used to measure physical parameters associated with functional ability [1]. This test is designed for people over 60 years of age, and consists of a six-item test:

- 8-Foot Up and Go—to assess agility/dynamic balance. Number of seconds required to get up from the chair, walk a distance of 8 feet (2.44 m), turn, and return to the seated position.
- 30-Second Chair Stand—to assess lower body strength. Number of full stands in 30 s with arms folded across the chest.
- Arm Curl—to assess upper body strength. Number of full curls in 30 s holding a hand weight of 2 kg.
- Back Scratch—to assess upper body flexibility. With one hand reaching over the shoulder and the other hand in the middle of the back. The result was the number of centimeters between extended middle fingers (+ or −).
- Chair Sit and Reach—to assess lower body flexibility. Test was performed in a sitting position in front of a chair, with one leg extended and hands reaching as far as possible toward toes. The result of this trial was the distance calculated in centimeters (+ or −) between extended fingers and tip of the toes.
- 2-minute Step Test—This test was used as alternate aerobic endurance test because of space limitation

Additionally, the hand grip strength was measured using the hand-held hydraulic dynamometer JAMAR (Model J00105, Lafayette Instrument Company). The measurement was taken in the standing position with a fully extended elbow. For analysis the best result (in kg) obtained from three reps was selected. 

### 2.4. Quality of Life Assessment

Quality of life was assessed using a short form of the standardized the World Health Organization Quality of Life questionnaire WHOQOL-bref in a Polish version. This questionnaire contains 26 questions and allows for recognition of the individual’s perception of quality of life in four domains [29]:

1. Physical health (activities of daily living, dependence on medicinal substances and medical aids, energy and fatigue, mobility pain and discomfort, sleep and rest, work capacity);
2. Psychological (bodily image and appearance, negative and feelings, self-esteem, thinking, learning, memory and concentration);
3. Social relationships (personal relationships, social support, sexual activity);
4. Environment (financial resources, freedom, physical safety and security, health and social care—accessibility and quality, home environment, opportunities for acquiring new information and skills, participation in and opportunities for leisure activities, physical environment, transport).

The questionnaire was self-administrated by participants. Domain scores were scaled in a positive direction (higher scores denote higher quality of life). The scores from questionnaire were calculated according to WHOQOL manual and the raw scores were converted to a scale of 1–100. 

### 2.5. Statistical Analysis

Basic features of the data in this study were described using: statistical means (M) and standard deviations (SD) for quantitative data, and frequencies or percentages for qualitative data. The differences in the scores obtained in the Senior Fitness Test battery, hand-grip strength and quality of life between women who met and did not meet the physical activity recommendations were identified using non-parametric Mann–Whitney U test. The association between meeting PA recommendations and the percentage of women with functional fitness assessed in the range of normal values for the age were assessed using Fisher’s exact test. 

All analyses were performed using IBM SPSS 20 software (IBM Corp. Released 2011. IBM SPSS Statistics for Windows, Version 20.0. Armonk, NY, USA). The level of significance of *p*-value was set at 0.05.

## 3. Results

The women who met physical activity (PA) recommendations achieved better results in all functional tests except a 2-min step trial (Table 2). The most significant differences were found in dynamic balance assessed by 8-foot Up and Go test. The physically active women needed less time to perform this trial (*p* < 0.001). The upper body strength was significantly higher among women who met PA recommendations in comparison to women who were insufficiently active. The significant differences were found in both the arm curl (*p* < 0.004) and hand-grip (*p* < 0.010) strength test.

According to the Senior Fitness Test (SFT) manual, the results of senior fitness test were compared to the normal range of scores for women in various age groups. It should be noted that regardless of physical activity level, the functional fitness of examined women was good. In the majority of trials more than 50% of women achieved scores that can be considered as average or above average for their age (Table 3). The most limitations in functional fitness were found in the flexibility of the upper body (back scratch test). Less than 50 % of women presented at least an average level of flexibility (42.6% of women who did not meet PA recommendations and 47.1% of women who met PA recommendations).

The analysis of quality of life in various domain depending on level of physical activity showed that senior women who met the physical activity recommendations achieved better quality of life. The mean scores in all four domain were higher among women who were physically active in comparison to women who did not meet PA guidelines. However, significant differences were found only in the social relationships domain (Table 4). The mean score in this domain was 70.63 ± 17.11 among women with a sedentary lifestyle, and women with the recommended level of physical activity achieved a score of 74.88 ± 14.04. General quality of life was also higher among physically active women (69.14 ± 8.75) in comparison to less active senior women (67.15 ± 10.99). However, this difference was not statistically important.

## 4. Discussion

The main purpose of this study was to identify the differences in functional capacity and quality of life among women over 60 years of age depending on the Global Recommendations on Physical Activity for Health. In our study 49% of women accumulated during the week the recommended dose of moderate or vigorous physical activity. According to the previous study, this result should be considered very optimistic. In the study performed by Shiroma, et al. [30] the percentage of older women who accumulated at least 150 min/week of moderate or vigorous physical activity, on the basis of measurement with the same cut-points settings (2690 cpm) was only 19.3%. However, participants in this study were volunteers from centers and universities for seniors. These types of institutions implement educational and promotional programs of health activities, which might have resulted in better health behaviors of participants.

In our study women who met physical activity recommendations achieved better results in senior fitness tests, especially in dynamic balance and upper body strength. The relationship between physical activity and functional fitness among older adults was confirmed in other studies [20,31,32]. In our study also the hand-grip strength was higher (2.12 kg) among women who met the PA recommendations. The similar results were reported by Laddu et al. [33]. In their research, the high PA groups (≥1200 metabolic equivalent (MET)-min/week) had stronger grip strength, more chair stands, and faster gait speeds than sedentary women.

Our study demonstrated that level of physical activity is significantly associated with social relationships domain of quality of life (QoL). However, regardless of domain, slightly better scores of QoL were identified among physically active women in comparison to insufficiently physically active participants. The positive influence of physical activity on quality of life was presented in many other studies [34,35,36,37,38,39]. Vagetti et al. [23] indicated that weekly volume of moderate and vigorous physical activity (MVPA) was associated with several domains of QoL, and higher frequency of MVPA was associated with better scores in 10 QoL domains.

In another study performed with the Polish population by Puciato et al. [40] using the WHOQOL-bref questionnaire, the odds of high assessment of overall quality of life increased with respondents’ physical activity level. However, in contrast to our study, physical activity was estimated using the International Physical Activity Questionnaire (IPAQ), while we measured physical activity by tri-axial accelerometers.

Participants in our study were recruited from centers and universities for seniors. The relationship between levels of physical activity and quality of life among students of universities for seniors was recognized by Krzepota, et al. [41]. They showed that highly active seniors declared better QoL in the psychological and social domains more often than other respondents. However, also in this study the physical activity was estimated using IPAQ questionnaire. 

The most significant relationships between meeting of physical activity recommendations and quality of life were found in the social relationships domain. This domain was also significantly related to physical activity level in other studies [36,41].

### Strength and Limitation of the Study

The main strength of this study is the use of accelerometers for identification of physical activity levels. This guarantees greater accuracy of the results in comparison to self-reported physical activity. The main limitation of this study is cross-sectional design with a convenience sample. Therefore, future studies should focus on the evaluation of the effects of implemented physical activity programs on functional fitness and quality of life among older adults. 

## 5. Conclusions

There is an association between meeting physical activity recommendations and functional fitness and quality of life among women over 60 years of age. Physical activity programs developed on the basis of WHO recommendations have the potential to improve functional capacity and quality of life among aging women. However, further experimental studies in this area are needed.

## Figures and Tables

**Table 1:** Participant characteristics.

Variables		*n*	%
Age (years)			
	60–64	70	32.9
	65–69	74	34.7
	70–74	43	20.2
	75–79	17	8.0
	80–85	9	4.2
	Total	213	100.0
BMI			
	18–24.9	53	24.9
	25–29.9	73	34.3
	30–34.9	56	26.3
	35–39.9	23	10.8
	40–45	8	3.8
	Total	213	100.0
Physical Activity (min/week)			
	MPA > 150	104	49.1
	MPA > 300	72	33.8
	VPA > 75	3	1.4
PA Recommendations			
	Met	105	49.3
	Not met	108	50.7

BMI—body mass index, PA—physical activity, MPA—moderate physical activity, VPA—vigorous physical activity.

**Table 2:** Functional fitness of women who met and did not meet the physical activity (PA) recommendations.

Fitness tests	Physical Activity Recommendations				*p*
	Not Met (*n* = 108)		Met (*n* = 105)		
	Mean	SD	Mean	SD	
8-Foot Up and Go (seconds)	7.01	2.19	6.20	1.25	**0.001**
Chair stand (No. of stands)	14.36	3.27	14.92	3.59	0.162
Arm Curl (No. of reps)	16.04	4.03	17.87	3.76	**0.004**
Chair Sit-and-Reach (cm +/−)	5.49	9.99	4.52	8.43	0.418
Back Scratch (cm +/−)	−8.94	12.53	−6.77	10.53	0.332
2-Min Step (number of steps)	87.31	25.06	82.61	25.89	0.164
Hand-Grip Strength (kg)	22.87	5.05	24.99	5.60	**0.010**

SD—standard deviation, Statistically significant differences at *p* ≤ 0.05 are given in bold.

**Table 3:** Results of the Senior Fitness Test (SFT) according to norm for the age among women who met and did not meet the physical activity recommendations.

Senior Fitness Test	Normal Ranges	Physical Activity Recommendations		*p*
		Not Met (*n* = 108)	Met (*n* = 105)	
8-Foot Up and Go (seconds)	Below	40.7%	38.5%	0.780
	Average/Above	59.3%	61.5%	
Chair stand (number of stands)	Below	10.2%	13.5%	0.526
	Average/Above	89.8%	86.5%	
Arm Curl (number of reps)	Below	13.9%	3.8%	**0.015**
	Average/Above	86.1%	96.2%	
Chair Sit and Reach (cm +/−)	Below	20.4%	15.4%	0.375
	Average/Above	79.6%	84.6%	
Back Scratch (cm +/−)	Below	57.4%	52.9%	0.581
	Average/Above	42.6%	47.1%	
2-Min Step (number of steps)	Below	25.9%	33.7%	0.232
	Average/Above	74.1%	66.3%	

Statistically significant differences at *p* ≤ 0.05 are given in bold.

**Table 4:** Quality of life (QoL) in various domains depending on meeting of physical activity recommendations.

Domains of Quality of Life (QoL)	Physical Activity Recommendations				*p*
	Not Met		Met		
	Mean	SD	Mean	SD	
Physical health	59.94	12.43	61.12	8.63	0.567
Psychological	64.23	12.58	65.81	11.41	0.476
Social relationships	70.63	17.11	74.88	14.04	**0.038**
Environment	73.81	12.62	74.76	11.77	0.714
QoL general	67.15	10.99	69.14	8.75	0.273

Statistically significant differences at *p* ≤ 0.05 are given in bold.
